# The Use of Extrinsic Performance Feedback and Reward to Enhance Upper Limb Motor Behavior and Recovery Post-Stroke: A Scoping Review

**DOI:** 10.1177/15459683241298262

**Published:** 2024-12-11

**Authors:** Dimitrios J. Palidis, Zoe Gardiner, Amelia Stephenson, Kevin Zhang, Jill Boruff, Lesley K. Fellows

**Affiliations:** 1 Department of Neurology and Neurosurgery, Montreal Neurological Institute, McGill University, Montreal, QC, Canada; 2 Schulich Library of Physical Sciences, Life Sciences, and Engineering, McGill University, Montreal, QC, Canada

**Keywords:** stroke rehabilitation, feedback, reward, gamification, virtual reality, motor skills

## Abstract

**Background:**

During post-stroke motor rehabilitation, patients often receive feedback from therapists or via rehabilitation technologies. Research suggests that feedback may benefit motor performance, skill acquisition, and action selection. However, there is no consensus on how extrinsic feedback should be implemented during stroke rehabilitation to best leverage specific neurobehavioral mechanisms to optimize recovery.

**Objective:**

To identify the existing evidence and research gaps regarding the effects of extrinsic feedback on upper extremity motor function in stroke survivors, and to map the evidence onto neurobehavioral concepts of motor performance, motor learning, and action selection.

**Methods:**

The MEDLINE, PsychInfo, EMBASE, and CINHAL databases were searched for relevant articles. A sequential screening process and data extraction were performed by 2 independent reviewers, and the results were reported according to the Preferred Reporting Items for Systematic Reviews and Meta-Analyses for Scoping Reviews guidelines.

**Results:**

A total of 29 studies were identified that met the criteria for inclusion. Beneficial effects of feedback were reported for clinical outcomes of rehabilitation interventions as well as motor performance, motor learning, and action selection post-stroke. Three studies showed that the addition of rewarding elements to positive performance feedback benefited learning or recovery.

**Conclusions:**

Extrinsic feedback has the potential to improve outcomes of stroke rehabilitation through effects on motor performance, motor learning, or action selection. To understand how these specific neurobehavioral processes contribute to recovery, clinical trials should include more granular behavioral measures. Rewarding feedback may be particularly beneficial, but more research is needed regarding the specific implementation of feedback.

## Introduction

Sensory feedback about the execution and results of our actions plays an essential role in the control of movement. Interventions that provide extrinsic feedback can improve the performance and learning of motor skills in healthy people, and could potentially facilitate relearning of skilled movements following stroke.^1,2^ Virtual reality, robotics, and motion tracking technologies are commonly used to provide extrinsic feedback during rehabilitation.^3,4^ There is a growing interest in using “gamification” to improve outcomes of stroke rehabilitation, in part by promoting engagement and motivation through feedback.^5^ However, there is no consensus regarding how extrinsic feedback should be implemented during stroke rehabilitation, nor what specific effects of feedback should be targeted. These questions may be usefully informed by neuroscientific and behavioral research which has characterized the powerful ways in which feedback shapes motor behavior and neural plasticity.

Work in healthy populations has shown that motor behavior and neuroplasticity are shaped by multiple, distinct mechanisms that are sensitive to specific types and parameters of feedback. It has long been recognized that extrinsic feedback about the size and direction of movement errors can guide learners to improve their performance through error correction.^6^ Recent evidence has demonstrated that motor performance and learning are also influenced by rewarding feedback and punishment in response to task successes and failures.^7,8^ Feedback conveying information about movement errors, rewards, and punishment each drive distinct forms of learning and neuroplasticity.^9,10^ and are sensitive to specific parameters of feedback including sensory modality,^11,12^ timing,^13,14^ frequency,^15,16^ valence,^17^ and motivational incentives.^18-20^ The specific characteristics of extrinsic feedback delivered during neurorehabilitation may therefore substantially affect its clinical benefits.

The effects of extrinsic feedback may differ for motor performance, motor learning, and action selection, with each component process contributing in distinct ways to stroke rehabilitation. Effects on *motor performance* refer to immediate improvements in movement execution upon the introduction of feedback. Feedback is said to benefit *motor learning* if improved performance during practice is retained over time in the absence of continued feedback.^21^ Feedback could improve motor performance, motor learning, or both during rehabilitation either by guiding stroke survivors to correct movement errors, or by motivating an increase in effort through reward or punishment.^1,22^ Rewarding feedback has also been shown to increase the retention of motor learning by improving the consolidation of motor memories.^18,23,24^

Feedback can also influence the voluntary selection of particular movements or movement patterns among alternatives, which we refer to as *action selection*. Following stroke, survivors tend to compensate for motor impairments by adapting their action selection. Stroke survivors tend to increase the use of their less affected upper extremity while neglecting to use their more impaired arm and hand.^25^ Compensation also occurs at the level of movement kinematics. For example, stroke survivors tend to use trunk displacement to transport their affected hand in order to compensate for impaired shoulder and elbow function.^25^ These compensatory movement strategies are thought to limit recovery by promoting the disuse of impaired movements instead of their improvement through continued practice.^25^ Extrinsic feedback could contribute to recovery by encouraging survivors to improve impaired movement patterns through practice instead of selecting alternative (compensatory) actions.

Here, we perform a scoping review with the objective of identifying the existing evidence and knowledge gaps regarding the effects of extrinsic feedback on upper extremity motor function in stroke survivors. In order to identify evidence specific to the effects of feedback, we consider studies that included multiple conditions in which stroke survivors performed similar motor tasks but which differed in the feedback that was provided. We map the effects of feedback onto distinct neurobehavioral concepts of motor performance, motor learning, and action selection in order to gain a more specific understanding of how extrinsic feedback may benefit stroke rehabilitation. We further characterize the use of extrinsic feedback according to sensory modality, timing, frequency, valence, and motivational incentives, as these factors have been shown to influence the effects of feedback on motor behavior. Previous reviews covering the effects of extrinsic feedback on upper limb motor function post-stroke are more than 10 years old.^26,27^

## Methods

A protocol for this review was constructed according to the guidelines of the Preferred Reporting Items for Systematic Reviews and Meta-Analyses (PRISMA) extension for scoping reviews and registered through publication on the Open Science Framework (https://doi.org/10.17605/OSF.IO/C625E).

### Eligibility Criteria

We included peer-reviewed journal articles written in English that meet the following criteria: (1) The participants included adult stroke survivors (greater than 18 years of age). (2) The participants performed a task or therapy involving the more affected upper extremity, which included the provision of extrinsic feedback contingent on task success or motor performance relative to a specific goal. Interventions that distorted visual or proprioceptive feedback in order to modify the magnitude of movement errors were not considered. We direct the reader to Israely and Carmeli^28^ and Liu et al^29^ for recent reviews specifically covering the effects of such error augmentation and error reduction paradigms in upper limb stroke rehabilitation. (3) At least 2 experimental groups or conditions were tested, in which the provision of feedback was different but participants otherwise performed equivalent interventions. (4) Clinical or behavioral outcomes were measured regarding motor function of the more affected upper extremity.

### Information Sources and Search Strategy

A health sciences librarian developed the search strategy and performed the literature searches in MEDLINE (Ovid), Embase (Ovid), PsycInfo (Ovid), and CINAHL (EBSCO) on December 21, 2022. No date limit or language limit was applied. The MEDLINE strategy was developed with input from the project team and adapted for use in the other databases. The complete search strategies are available in an institutional data repository: https://doi.org/10.5683/SP3/ADFAGD.

### Selection of Sources of Evidence

The search results were exported to Endnote software for deduplication, and exported to Rayyan software for screening. Articles were selected for inclusion through a sequential screening process in which 2 reviewers independently examined first the titles and abstracts, then the full texts of each record. All conflicts between reviewers were resolved through discussion.

### Data Charting Process

Data extraction was performed through a standardized data charting form using Covidence software. Data extraction for each article was performed independently by 2 reviewers, and all discrepancies in the results were resolved through discussion.

### Data Items

The following items were extracted for each selected study: (1) Study design and sample size; (2) participant characteristics: age, sex, stroke type, duration, lesion location, and cognitive and motor impairment; (3) study intervention: motor task or therapy performed, number of movements performed or time on task; (4) Feedback characteristics: movement variables conveyed by feedback, timing (concurrent to movement, terminal, or summary), valence (feedback stimuli delivered for good versus bad performance), sensory modality, frequency, elements of additional motivational salience (eg, monetary reward, complex audiovisual stimuli, and social comparison); and (5) study results (mean values or differences on upper limb motor outcomes by condition).

### Synthesis of Results

Extracted data items were summarized in table format, along with a descriptive overview of the results. The tables and summary were organized to separately present findings related to clinical outcomes, motor performance (immediate changes in motor function in response to feedback), motor learning (changes in motor performance measured in the absence of feedback following repeated practice of a specific task), and action selection (increased use of the affected upper extremity or decreased use of compensatory movement patterns).

## Results

**Figure 1.**
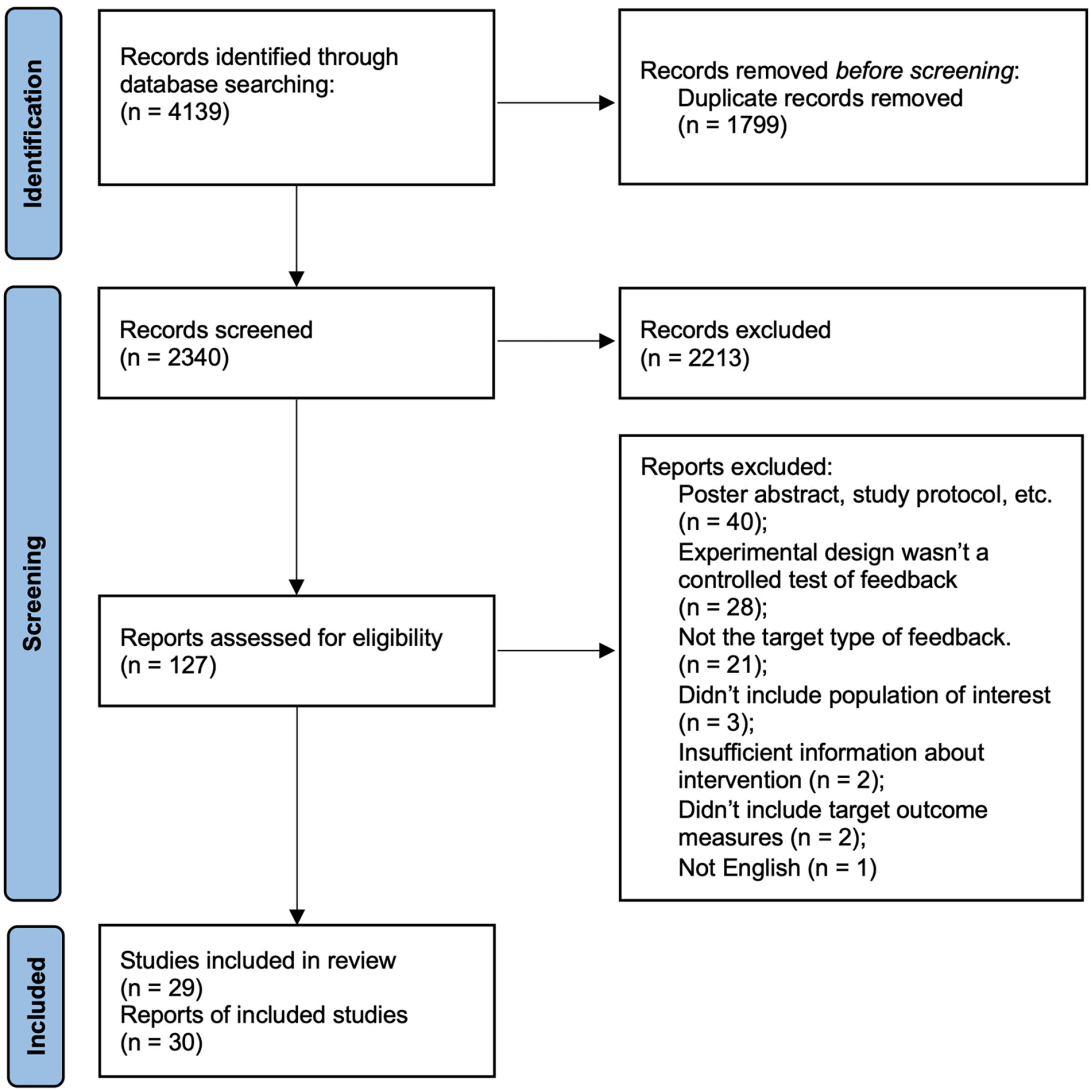
Screening and review flowchart.

The searches returned 4139 records. A total of 1799 duplicates were removed. A total of 2213 records were eliminated during initial screening of titles and abstracts. A total of 127 full texts were reviewed, 97 studies were found not to meet the inclusion criteria, leaving 30 included reports. Two publications were grouped as a single study for reporting as they described analyses of different outcomes from the same dataset.^30,31^ Figure 1 shows the PRISMA flowchart of the search and screening process.

### Study Characteristics

We identified 15 studies with between-group designs comparing the effects of different feedback conditions. Thirteen of these studies were randomized controlled trials (RCTs), including 5 pilot studies.^30-43^ Eleven RCTs involved repeated rehabilitation sessions in clinical settings, while 2 tested the use of feedback from wearable devices during daily life.^34,39^ Two studies compared the effects of different feedback conditions on motor learning over multiple practice sessions, but did not measure clinical outcomes and were not designated as RCTs.^44,45^ The total sample size of studies with between-group designs ranged from N = 11 to 45.

Thirteen studies used within-participants designs to assess short-term (within a single session) changes in movement outcomes under different feedback conditions.^46-58^ Six of these studies were designated crossover design RTCs, including 2 pilot studies.^49-52,56,57^ Two studies were case series with sample sizes of n = 2 and 5.^46,48^ One study tested a clinical rehabilitation program under different feedback conditions using a within-participants reversal design involving 90 sessions.^59^ The sample sizes of studies using within-participants designs, excluding case series, ranged from N = 5 to 43.

### Clinical Characteristics of Participants

#### Stroke-Induced Lesions

Two studies only included participants with ischemic stroke,^48,53^ 14 included participants with both ischemic and hemorrhagic stroke,^32,34,35,39-41,43,44,52,55-59^ and 13 did not specify the type of stroke.^30,31,33,35,36,38,42,45,46,49-51,54^ Twenty-two studies did not specify the vascular territory of stroke. Studies that did describe the affected vascular territories included patients with middle cerebral artery,^41,48,49,53,58^ anterior cerebral artery,^58^ anterior circulation,^34,59^ posterior circulation,^34^ and lacunar^34,59^ strokes. Seven studies in-cluded patients with both cortical and subcortical lesions,^30,31,33,41,42,44,48,58^ and the remaining 22 studies did not report whether lesions were cortical or subcortical. No study investigated whether the extent, location, or type of lesions were related to the effects of feedback.

#### Stroke Duration

Four studies included patients with acute stroke (duration less than 2 weeks),^34,37,49,51^ 12 studies included patients with early subacute stroke (duration between 2 weeks and 3 months),^32,34,37,38,43,48,49,51-53,55,58^ 10 studies included patients with late subacute stroke (duration between 3 and 6 months),^30-32,37,38,43,46,51,52,55,58^ and 20 studies included patients with chronic stroke (duration greater than 6 months).^30,31,33,35,36,38-42,44,47,48,50-54,56,57,59^ One study did not specify the duration of stroke.^45^ No study analyzed whether stroke chronicity was related to outcomes.

#### Cognitive Impairment

Twelve studies excluded participants with cognitive function below a threshold determined by Mini Mental Status Examination (MMSE) or Montreal Cognitive Assessment (MoCA) tests.^32,33,35-37,40,44,46,49,52,53,59^ Thirteen studies excluded participants with aphasia,^32,35,36,40-43,45,49,51,52,58,59^ and 9 studies excluded participants with neglect.^35,37,44-46,52-54,59^ Only 2 studies included measures of cognitive function in their analysis. Cirstea et al^31^ reported that greater cognitive impairment in verbal memory, planning, and mental flexibility were associated with worse motor learning.^31^ This association was only present for learning mediated by verbal feedback regarding their upper limb movement patterns, but not learning through visual feedback regarding movement accuracy. Quattrocchi et al^44^ controlled for cognitive function by including cognitive test scores as covariates in their statistical analyses but did not report on the effects of these variables.

### Effects of Feedback

#### Clinical Outcomes

Nine studies reported statistically significant effects of feedback on clinical outcome measures (Table 1). According to the international classification of Functioning, Disability and Health model (ICF), 8 studies reported effects at the level of body functions and structures,^32,33,36,37,40,42,43,59^ 7 at the level of activities,^32,33,37,38,40,43,59^ and 3 at the level of participation.^32,40,59^

**Table 1.** Effects of Feedback on Clinical Outcomes.

Study: (citation; design)	Participants: (sample size; age; sex)	Stroke: (stroke type; lesion location; chronicity)	Baseline impairment: (motor and cognitive scores, exclusion criteria)	Intervention: (description; dose)	Feedback: (description; timing; frequency; valence)	Results: (statistically significant effects of feedback)
Bang^32^; Pilot RCT comparing feedback (fb) vs no feedback (ctr)	feedback group: n = 10 age: 61 control group: n = 10 age: 58; men and women	Ischemic and hemorrhagic; location unspecified; early and late subacute	FM-UE: 34.7 (fb), 35.8 (ctr); Extension ≥ 10° at MCP, IP, and wrist joints; MMSE ≥ 24; Exclusion: spasticity, aphasia	Functional task training and household activities while wearing a mitt to constrain less affected hand; Dose: 20 h therapy + 100 hr wearing constraint at home during 4 wk	Auditory feedback tone when trunk moved away from chair back; Timing: concurrent; Frequency: 100%; Valence: negative	ARAT change: Fb (+14.2) > ctr (+10.1) FM-UE change: fb (+14.8) > ctr (+9.5)^a^ mBI change: fb (+22.4) > ctr (+15.1) MAL-AOU change: fb (+1.13) > ctr (+0.81)
Carey et al^33^; RCT comparing feedback (fb) vs no feedback (ctr)	feedback group: n = 10 age: 66 [55-79] control group: n = 10 age: 67 [54-85]; men and women	Type unspecified; cortical and subcortical; chronic	BBT: 23.9 (fb), 31.7 (ctr); JTT: 217 s (fb), 145 s (ctr); Extension ≥ 10° at MCP joint; MMSE ≥ 25	Repeated Extension/Flexion movements at MCP joint and wrist; Dose: 1800 trials over 10 d	Visual cursor, accuracy score, and intermittent instructions (fb); Timing: concurrent (cursor), terminal (score, instructions); Frequency: 100% (cursor, score), faded (instructions); Valence: neutral	BBT no. of blocks change:fb (+2.0) <ctr (+4.9) Finger ROM change: fb (+22°) > ctr (+1.2°)
Colomer et al^59^; A-B-A reversal study comparing feedback (fb) vs no feedback (ctr),	N = 30; Age = 58; Men and women	Hemorrhagic and ischemic (anterior and lacunar circulation); chronic	FM-UE: 50.2; MMSE > 23; Exclusion: Spasticity, Aphasia, neglect	Exercises were a variety of tabletop unimanual tasks focused on elbow, wrist, and MCP joint. Some involved object manipulation. Dose: 30 45-min sessions per condition	Visual and auditory feedback and scoring in mixed reality indicating if tasks were performed successfully or not depending on speed, accuracy, compensation; Timing: terminal; Frequency: 100% Valence: positive, negative;	Ctr improved: MAL-AOU (+5.3)^b^; Fb improved: MAL-AOU (+17.8)^b^; MAL-QOM (+18.1)^b^; WMFT-TIME (−4.1 s)^a^; BBT no. of blocks (+2.5); NHPT time (−9.5s);
Fluet et al^35^; Pilot RCT comparing feedback with enhanced motivation (EM) vs unenhanced control (UC)	UC group: N = 6; Age = 65; EM group: N = 5; Age = 58; Men and women	Unspecified type and location; Chronic	FM-UE: 51 (UC), 44 (EM); MoCA ≥ 22; Exclusion: Neglect, aphasia	Exercises involved single- and multi-joint movements of the arm and hand to control visual simulations using a motion tracking device in home environment; Dose: 20+ min/d for 12 wk	Scoring in simulation depended on movement speed, and ROM. In EM condition only, changes in graphics and scoring opportunities corresponded with task progression; Timing, frequency, and valence unspecified.	No statistical comparisons performed.
Henrique et al^36^; RCT comparing feedback (fb) vs no feedback (ctr)	feedback group: n = 16 age: 76 control group: n = 15 age: 76; men and women	Unspecified type and location; Chronic	FM-UE: 16.6 (fb), 20.3 (ctr); MMSE ≥ 19; Exclusion: aphasia, spasticity	Participants performed multi-joint arm movements involving the shoulder, elbow, and wrist while standing; Dose: 24 30-min sessions over 12 wk	Visual and auditory feedback in non-immersive VR about range of motion and accuracy; Timing: terminal; Frequency: 100%; Valence: pos. and neg.	FM-UE change: Fb (+14.7) > ctr (+9.1)^a^ FM-shoulder, elbow, forearm change: Fb (+10.6) > ctr (+5.5)^b^
Park et al^37^; RCT comparing feedback (fb) vs no feedback (ctr)	feedback group: n = 22 age: 57 control group: n = 21 age: 62; men and women	Hemorrhagic and ischemic; unspecified location; acute and subacute	Manual function test (max: 32): 10.8 (ctr), 12.9 (fb); MMSE > 24; Grip power < 30 kg; Exclusion: Neglect	Participants performed isometric and isotonic resistance exercises for the hand. In Fb condition, feedback was delivered from a force sensor. Dose: 30 min/session for 30 sessions	Visual-auditory feedback indicated whether the target amount and duration of force was successfully produced the form of a game. Timing: Concurrent; Frequency: 100%; Valence: unspecified	Grip strength change (kg): Fb (+6.2) > ctr (+3.8)BBT no. of blocks change: Fb (+7.0) > ctr (+4.2) MFT change: Fb (+3.3) > ctr (+1.1)
Popovic et al^38^; Pilot RCT comparing feedback (fb) vs no feedback (ctr)	feedback group: n = 10 age: 58 [38-68] control group: n = 10 age: 57 [38-72] men and women	Unspecified type and location; early and late subacute, chronic	FM-UE: FB: 35 [24-51], CTR: 34 [27-42];	Planar reaching movements to attain targets or follow trajectories while holding the handle of a manipulandum; Dose: 15 sessions each 15 min	Visual-auditory feedback of number of targets attained and movement speed and accuracy. Performance was tracked on high score list; Timing: terminal; Frequency: 100%; Valence: positive, neutral	mDT smoothness post/pre: Fb (1.4) > ctr (1) mDT speed post/pre: Fb (1.2) > ctr (0.8) Task time/session (min): Fb (14.6) > ctr (11)
Shin et al^40^; RCT comparing feedback (FB) vs no feedback (CTR)	feedback group: n = 19 age: 57 control group: n = 14 age: 60 men and women	Hemorrhagic and ischemic; unspecified location; mean chronicity: 15 months (CTR), 13.6 months (FB)	FM-UE: 48.2 (CTR), 53.4 (FB); Exclusion: Aphasia, MMSE < = 24	Singe joint and complex hand and wrist movement exercises; Dose: 20 30-min sessions over 4 wk	Visual-auditory performance feedback in gamified simulations depended on factors such as speed and range of motion depending on task; Timing, frequency and valence unspecified;	FM-UE change post training:Fb (+4.9) > ctr (+1.4)FM-UE change at follow up: Fb (+5.3) > ctr (+1.3)JTT change post training: Fb (+10.3) > ctr (+3.5)^b^ JTT change at follow up: Fb (+10.9) > ctr (+3.8)^b^ SIS 3.0 total score change post training: Fb (+53.1) > ctr (−2.1)
Thielman^42^; Pilot RCT comparing feedback (FB) vs trunk restraint (TR)	FB group: n = 8 age: 63 [51-72] TR group: n = 8 age: 63 [54-75]; men and women	Unspecified type; cortical and subcortical; chronic	FM-UE: FB: 34.1 [20-33] TR: 30.4 [22-38]; Exclusion: Aphasia, apraxia	Tabletop exercises while seated for 3D reaching, grasping, and functional task training. The TR group wore a harness restraining trunk movement. Dose: 12 sessions 40 to 45 min long involving 150 to 200 repetitions	In the FB condition, an auditory signal indicated when the back was not in contact with the chair due to forward trunk movement. Timing: concurrent; Frequency: Faded; Valence: negative	RPS near target change: FB (3.3) > TR (0.9)
Widmer et al^43^; RCT comparing performance feedback plus reward (REW) versus performance feedback (CTR)	REW group: n = 19 age: 63 CTR group: n = 18 age: 66; men and women	Ischemic; location unspecified; early and late subacute	FM-UE: 32.1 (REW), 32.8 (CTR); MMSE: 28 (REW), 28 (CTR); Exclusion: Aphasia	3D reaching movements controlled a cursor on a visual display in order to catch falling targets. The difficulty was adapted iteratively. Dose: 15 1-h sessions.	CTR: visual targets disappear with a 1 s delay after being touched. REW: hits and misses result in immediate gamified audio-visual feedback linked to scoring and monetary reward. Timing: terminal; Frequency: 100% Valence: positive, negative	FM-UE change at 3-month retention: REW > CTR (diff: 8.2)^a^ WMFT-SCORE change at 3-month retention: REW > CTR (diff: 0.63)^a^ BBT change post training: REW > CTR (diff: 6.0) BBT change at 3-month retention: REW > CTR (diff: 9.7)

Abbreviations: RCT, randomized controlled trial; ARAT, action research arm test (0-57 scale, minimum clinically important difference (MCID), 12-17^60^); FM-UE, Fugyl-Meyer Upper Extremity Score (0-66 scale, MCID, 4.24-7.25^61^); MMSE, mini mental status examination (0-30 scale); mBI, Modified Barthel Index (1-100 scale, MCID, 9.25^62^); MAL-AOU, motor activity log, amount of use subscale (0-5 scale, MCID, 0.5^63^); MAL-QOM, motor activity log, quality of movement subscale (0-5 scale, MCID, 1.0-1.1^60^); BBT, box and blocks test (number of blocks moved/min); ROM, range of motion; grip strength, grip force measured by dynamometry (MCID, 5-6.2 kg^60^); WMFT-TIME, wolf motor function test performance time (max, 120 second, MCID, 1.5-2 s reduction^64^); WMFT-SCORE, wolf motor function test score (0-6 scale, MCID, 0.2-0.4^64^); NHPT, 9 hole peg test (time to complete test in seconds); MFT, manual function test (0-32 scale); MoCA, Montreal Cognitive Assessment (max, 30); mDT, modified drawing test (speed and smoothness metrics are scored from 2D kinematics); JTT, Jebsen–Taylor Test (Shin et al. used a 0-105 nonstandard scoring scale^40^); SIS 3.0, Stroke Impact Scale version 3.0 (sum of 0-100 score on 8 domains); RPS, Reaching Performance Scale (0-18 scale scored for both a near and far reaching target).

a Differences between groups exceeding an established MCID.

b Use of nonstandard, unspecified scoring scales.

The types of movements performed in studies reporting effects on clinical outcomes reflected a large range of activities performed during rehabilitation. Movement types included 3-dimensional reaching,^36,42,43^ planar or linear reaching,^38,59^ isolated hand movements,^37,40,59^ reaching and grasping,^42^ single joint movements,^33,40,59^ and complex functional tasks (eg, pouring water, folding, and combing hair).^32,42^ The information conveyed by feedback generally reflected the specific goals of these varying tasks. Feedback was contingent on movement accuracy,^33,36,38,43,59^ speed,^38,40,41,43,59^ range of motion,^33,36,40^ upper limb kinematics,^59^ trunk kinematics,^32,42^ and force production.^37^

The effect size of clinical benefits due to feedback was inconsistent; we only identified 4 studies reporting benefits of feedback exceeding established thresholds for minimum clinically important differences (Table 1). However, estimates of minimal clinically importance differences were not available for several measures. The variability in the effects of feedback is likely due to the fact that the studies identified here were small and highly heterogeneous in their interventions and clinical outcome measures

#### Performance

Five studies reported effects of feedback on motor performance (Table 2). We defined performance effects as immediate improvements in movement execution upon the introduction of feedback. Unlike motor learning, performance effects do not necessarily persist after the removal of feedback.^21^ Four of the studies identified here used crossover designs with minimal washout periods, which should primarily measure transient effects of feedback.^49,51,54,55^

**Table 2.** Effects of Feedback on Motor Performance.

Study: (citation; design)	Participants: (sample size; age; sex)	Stroke: (stroke type; lesion location; chronicity)	Baseline Impairment: (motor and cognitive scores, exclusion criteria)	Intervention: (description; dose)	Feedback: (description; timing; frequency; valence)	Results: (statistically significant effects of feedback)
Cameirão et al^47^; experiment with crossover design comparing 3 feedback conditions	n = 5; age: 56.6 [52-62]; men and women	Unspecified type and location; Chronic	SIS physical domain: 74.4 [40.2-96.9];	Repeated elbow flexion-extension movements; Dose: 8 min of repeated movements per condition	Score indicating ROM and compensation (1), **or** score and audio-visual verbal encouragement (2), **or** score and movement mapped to game (3); timing: terminal; frequency: 100%; valence: positive, neutral;	No statistical analysis performed
Cruz et al^49^; RCT with crossover design comparing feedback (fb) vs no feedback (ctr)	Ctr condition first:N = 21; Age = 65; Fb condition first: N = 22; Age = 68; Men and women	MCA territory; Acute and early subacute	NIHSS motor: 2.5 (ctr), 3.0 (fb); Neglect frequency: 23% (ctr), 24% (fb); Exclusion: MMSE below unspecified cut-off, aphasia	Repeated hand-to-mouth movements; Dose: 1 session per condition averaging 2.9 min (range: 1-10),	Vibration stimulus delivered to arm if target movement amplitude or correct starting position was not achieved, or rate of movements was inadequate.; Timing: concurrent; Frequency: 100%; Valence: negative	Correct movements/min: fb (25.7) > ctr (18.5) Movements/min: fb (30.0) > ctr (24.7) Correct amplitude/min: fb (1495°) > ctr (1326°)Amplitude/min: fb (1258°) > ctr (1028°)
Durham et al^51^; Crossover RCT comparing feedback with internal (IF) versus external (EF) focus.	N = 42; Age = 63 (IF first);Age = 59 (EF first); Men and women	Unspecified type and location; Any stage	FM-UE: 45.6 (IF first), 42.1 (EF first); Exclusion: Aphasia	Tasks were reaching to grasp a jar (1), placing a jar forward on a Table (2), and placing a jar on an elevated surface (3); Dose: 16 reaches per condition and task.	Verbal feedback after each trial regarding movement speed, accuracy, smoothness, or joint kinematics. Statements chosen to induce either internal or external focus of attention; Timing: terminal; Frequency: 100%; Valence: neutral;	Time to peak velocity: Task 1: IF < EF (IF First: 22%, IF second: 27%, EF first: 29%, EF second: 26%)Movement duration: Task 2: IF > EF (IF first: 2.5 s, IF second: 2.4 s, EF first: 2.3 s, EF second: 2.1 s)Time to peak deceleration: Task 2: IF < EF (IF first: 37%, IF second: 37%, EF first: 41%, EF second: 44%)
Rizzo et al^53^; Repeated-measures experiment comparing hand position feedback (HND) versus hand plus gaze feedback (EYE-HND).	N = 13; Age: 58 [34-78]; Men and women;	Ischemic MCA territory; Early subacute and chronic;	FM-UE: 55.5 [30-66]; MMSE ≥ 24; Exclusion: Neglect;	Participants made 3D reaches to touch visual targets on a table while their finger and gaze positions were recorded; Dose: 152 reaches per condition.	In both conditions, visual feedback indicating the position of the fingertip at movement endpoint relative to the target. In EYE-HND, visual feedback also indicated the gaze position at the point of peak fingertip velocity; Timing: terminal; Frequency: 100%; Valence: neutral;	Saccade latency (ms): Fb (172) > ctr (82) Reaction time (ms): Fb (558) < ctr (600) Difference b/w saccade and reach onset (ms): Fb (386) < ctr (519) Reach duration (ms): Fb (537) < ctr (604) Saccade duration (ms): Fb (50) < ctr (60) Saccade amplitude (mm): Fb (53.9) > ctr (51.4 mm) Reach error (mm): Fb (14.6) < ctr (21.4)
Secoli et al^54^; Crossover experiment comparing feedback (fb) vs no feedback (ctr)	N = 14; Age: 56 [37-77]; Men and women;	Unspecified type and location; Chronic;	FM-UE excluding wrist and hand (max: 42): 25.9 [15-32]; Exclusion: Neglect, spasticity, apraxia	Cyclic 3D reaches controlled a cursor to track a visual target. A robot exoskeleton provided assistance depending on the error. The task was performed with and without visual distraction. Dose: 20 6-s repetitions in each condition.	Repeating auditory tones were played when the error between the cursor and target exceeded 1 inch. The frequency of tone repetition increased proportionally to the tracking error; Timing: concurrent; Frequency: 100%; Valence: negative;	Tracking error during visual distraction: FB < CTR; Vertical assistive force from robot during visual distraction: FB < CTR;
Simonsen et al^55^; Crossover experiment comparing feedback (fb) vs no feedback (ctr)	N = 11; Age: 64 [52-78]; Men and women;	Ischemic and hemorrhagic; Unspecified location; Early and late subacute	ARAT: 29.3 [20-39];	Planar reaching movements to trace a rectangular pattern on a horizontal visual display. Dose: 2 sessions, number of trials unspecified.	A visual cursor was displayed during the movement and the entire trajectory was shown after each trial. The color of the feedback varied depending on distance from target; Timing: concurrent, terminal; Frequency: 100%; Valence: positive, negative	Trajectory smoothness: FB > CTRTrial duration (S): FB (53.8) > CTR (27.8) Trajectory variability: FB < CTR
Zimmerli et al^58^; Crossover experiment comparing feedback with easy (EASY), balanced (BAL), or hard (HARD) performance criteria	N = 10; Age: 51 [24-73]; Men and women;	Ischemic (territories: MCA, ACA, and watershed) and hemorrhagic (basal ganglia); Cortical and subcortical; Early and late subacute	FM-UE: 39.4 [26-54]; ACE-R: 78.4; Exclusion: Aphasia	Planar reaching movements in a robotic exoskeleton controlled a visual cursor. Participants reached to varying locations and squeezed a hand grip to attain targets. Dose: 2 min of repeated trials per condition	A positive or negative auditory cue indicated if the target was attained within a time limit. The available time was either 0.5x (HARD), 1x (BAL), or 2x (EASY) the required time estimated for each participant. Timing: terminal; Frequency: 100%; Valence: positive, negative	Percent successful trials: EASY > HARD; No differences observed between conditions for the paretic arm in movement speed, hand closing time or reaction time.

Abbreviations: SIS, Stroke Impact Scale; MCA, middle cerebral artery; ACA, anterior cerebral artery; NIHSS, National Institute of Health Stroke Scale; MMSE, mini mental status examination; FM-UE, Fugyl-Meyer Upper Extremity Score (max, 66); ARAT, action research arm test; ACE-R, Addenbrooke’s cognitive examination-revised.

Cruz et al^49^ found that during a repetitive hand-to-mouth movement exercise, feedback dependent on the rate and amplitude of movements improved both of these factors, compared to no feedback. Durham et al^51^ compared verbal feedback regarding various movement features that was formulated to induce either an internal or external focus of attention during reaching and grasping tasks. For example, participants were told to “think about lifting their arm up higher” (internal) versus to “think about being higher off the table” (external). Feedback targeting an external focus of attention improved movement duration and measures related to the velocity and acceleration profiles of reaching. Rizzo et al^53^ found that the addition of feedback indicating gaze position during three-dimensional (3D) reaching improved movement accuracy, duration, and reaction time. Secoli et al^54^ had patients perform 3D arm movements to track a visual target with and without visual distraction. They found that auditory feedback dependent on tracking error ameliorated reductions in performance due to distraction. Simonsen et al^55^ found that visual feedback about the accuracy of 2D trajectory tracing improved the smoothness and variability of movements.

Overall, the existing literature has shown that feedback can lead to performance improvements in the speed, accuracy, and vigor of reaching movements, indicating that it could potentially increase movement quality during therapy. However, it isn’t clear whether the magnitude of these improvements is of clinical importance. Notably, Cruz et al^49^ reported that feedback increased the rate of movement repetitions by over 5 repetitions/minute, which could translate to hundreds of additional repetitions per session. The studies reviewed here focused almost entirely on proximal arm function during reaching; we didn’t identify any studies showing that feedback can lead to performance improvements other areas such as manual dexterity or the production of effort during resistance training.

#### Motor Learning

Four studies reported effects of feedback on motor learning, which we defined as improvements in task performance through repetitive practice that were assessed post-practice in the absence of feedback (Table 3). Three of these studies involved extensive training of 3D reaching movements. Maulucci and Eckhouse^45^ tested the effects of feedback indicating when the hand deviated from an ideal trajectory, compared to no feedback. They found that feedback resulted in improved adherence to the ideal trajectory and reduced hand oscillations. Cirstea and Levin,^30^ Cirstea et al^31^ found that feedback about extension of the shoulder and elbow joints led to improvements in the range of motion and coordination of these joints, while feedback about the location of the hand at movement endpoint led to improved endpoint precision. Subramanian et al^41^ had participants train with feedback that depended on movement speed, accuracy, and trunk displacement. Feedback was either delivered in a physical environment or was coupled with salient, gamified stimuli in virtual reality. They found that shoulder range of motion improved only when feedback was delivered in the virtual environment. Quattrocchi et al^44^ investigated the effects of feedback linked to monetary reward and punishment, compared to neutral feedback, when participants learned to compensate for mechanical perturbations during 2D reaching. Both reward and punishment enhanced learning, while only reward benefited the retention of learning.

**Table 3.** Effects of Feedback on Motor Learning.

Study (citation; design)	Participants: (sample size; age; sex)	Stroke: (stroke type; lesion location; chronicity)	Baseline Impairment: (motor and cognitive scores, exclusion criteria)	Intervention: (description; dose)	Feedback: (description; timing; frequency; valence)	Results: (statistically significant effects of feedback)
Chen et al^48^; Case series with crossover design comparing feedback (fb) versus no feedback (ctr)	N = 5; Age = 58 [52-64]; Men and women	Ischemic MCA territory, subcortical and cortical; Early subacute and chronic	FM-UE: 40 [30-53]; Shoulder flexion and elbow extension ≥ 10°	Forward 3D reaching movements from start position to target; Dose: 72 movements per condition	Consonant or dissonant auditory tone played depending if elbow, shoulder, and trunk angles followed an ideal trajectory; Timing: concurrent; Frequency: 100%; Valency: positive, negative	No statistical analysis performed
Cirstea^30,31^; RCT comparing knowledge of results (kr) versus knowledge of performance (kp) feedback types	KR group: N = 14; Age = 56; KP group: N = 14; Age = 59;Men and women	Type unspecified; Cortical and subcortical; Late subacute and chronic;	FM-UE: 45.8 (kr), 40.8 (kp);	Repeated 3D pointing movements from a starting position to target without vision; Dose: 750 repetitions over 10 1-h sessions.	Visual feedback about movement precision (kr) or verbal feedback about shoulder flexion and elbow extension (kp); Timing: terminal (kr), concurrent (kp); Frequency: intermittent (kr), faded (kp); Valence: neutral	KR improved: precision, TEMPA (+3.7) KP improved: movement time, segmentation, movement variability, shoulder horizontal adduction (+9.6°), shoulder flexion (+6.7°), inter-joint coordination, TEMPA (+3.7)
Maulucci^45^; Non-randomized experiment comparing feedback (fb) versus no feedback (ctr)	feedback group: n = 8 control group: n = 8; age: between 50 and 70 men and women	Unspecified type, location, and chronicity	Exclusion: Neglect, aphasia	Forward 3D reaching to touch 3 different targets at shoulder height; Dose: 24 reaches per session, 18 sessions over 6 wk	An auditory tone played if hand position deviated from an ideal trajectory. Timing: concurrent; Frequency: 100%; Valence: negative	FB improved: Endpoint accuracy, oscillations, adherence to ideal trajectory. CTR improved: Endpoint accuracy, movement speed.
Quattrocchi et al^44^; Randomized experiment comparing reward (REW), punishment (PUN), and neutral feedback (NEU).	Neutral group: n = 15 age: 59 Reward group: n = 15 age: 59Punishment group: n = 15 age: 56; men and women	Ischemic and hemorrhagic; Cortical and subcortical; Chronic	FM-UE: 41.8 (NEU), 49.8 (REW), 45.6 (PUN); Shoulder flexion ≥ 45° MMSE > 24; Exclusion: Neglect	Participants adapted to compensate for velocity-dependent mechanical perturbations during planar reaching. Retention of adaptation was tested after perturbations were discontinued; Dose: 2 sessions, each with 350 reaches with perturbation	All groups saw visual cursor indicating hand position relative to target. During first session only, REW and PUN received scores depending on trajectory error, indicating monetary gains or losses; Timing: terminal; Frequency: 100%; Valence: positive, negative, neutral	Adaptation: REW > NEU; PUN > NEU; Adaptation session 2 (no feedback): REW > NEU; PUN > NEU; Retention of adaptation: REW > NEU (sessions 1 and 2) REW > PUN (session 2)
Subramanian et al^41^; RCT comparing feedback in virtual (VE) vs physical (PE) environments	PE group: n = 16 age: 60 VE group: n = 16 age: 62; men and women	Hemorrhagic and ischemic; Cortical (MCA, Frontal) and Subcortical; Chronic	FM-UE: 42.1 (PE), 41.1 (VE); Exclusion: Aphasia, Apraxia,	3D reaches pointing toward 6 targets which were either numbered rectangles (PE) or objects in non-immersive VR (VE). Dose: 72 reaches per session, 12 sessions	“Ping” or “Buzz” sound if speed and accuracy criteria were achieved or not. “Woosh” sound if trunk displacement was ≥ 5 cm. In VE only, success was also indicated by visual animation and increasing point score; Timing: Terminal; Frequency: 100% Valence: Positive, negative	PE improved: elbow extension in severe subgroup at follow up (+11°) VE improved: Shoulder adduction at post (+9°), shoulder flexion at post and retention (+6°,+13°), elbow extension in mild subgroup at follow up (+24°)

Abbreviations: MCA, middle cerebral artery; FM-UE, Fugyl-Meyer Upper Extremity Score (max: 66); TEMPA, upper extremity performance test for the elderly (range: 0 [normal] to -69 [no function]); ROM, range of motion; MMSE, mini mental status examination (max: 30);

In summary, the studies reviewed here found that feedback can improve learning of reaching movements both in terms of endpoint control and in the kinematics of the shoulder and elbow joints, depending on which aspect of movements are conveyed by feedback. Moderate improvements were reported for shoulder adduction, shoulder flexion, and elbow extension due to training with feedback, ranging from 9 to 9.6°, 6 to 13°, and 24°, respectively.^30,31,41^ Effect sizes were unfortunately not reported for other motor learning outcomes. Studies also found that the addition of salient and rewarding elements to feedback can lead to additional benefits. As was the case for motor performance, there were no studies reporting effects of feedback on motor learning for tasks focused on distal arm function.

#### Action Selection

Five studies reported effects of feedback on action selection, which we defined as changes in the selection of particular movements or movement patterns (Table 4). Four studies reported that feedback resulted in immediate and short-term reductions in the use of compensatory movement patterns during repetitive, simple reaching tasks.^46,50,56,57^ Cai et al^46^ found that compensatory trunk movements were reduced by audio-visual feedback delivered in non-immersive virtual reality, compared to a baseline condition with no feedback. Douglass-Kirk et al^50^ tested the effects of musical feedback stimuli that stopped whenever compensatory movements of the trunk, shoulder, or scapula were detected. They found that feedback reduced compensation compared to a control condition with no feedback. Valdes et al^56^ compared the effects of visual versus haptic feedback dependent on trunk displacement. Both types of feedback led to similar reductions in compensatory trunk movements. Similarly, Valdes and Van der Loos^57^ found that combined haptic and visual feedback reduced trunk displacement, but that the addition of gamified scoring produced no additional benefits.

**Table 4.** Effects of Feedback on Action Selection.

Study: (citation; design)	Participants: (sample size; age; sex)	Stroke: (stroke type; lesion location; chronicity)	Baseline impairment: (motor and cognitive scores, exclusion criteria)	Intervention: (description; dose)	Feedback: (description; timing; frequency; valence)	Results: (statistically significant effects of feedback)
Cai et al^46^; Case series with repeated measures design comparing feedback (fb) versus no feedback (ctr)	n = 2;age = 65;66;men;	Unspecified types and locations; Late subacute;	FM-UE: 29;36; MMSE: 25;27; Exclusion: pain, spasticity, neglect	Repeated reaching movements with direction constrained by robot; Dose: 45 movements per condition	Auditory, Visual, and verbal feedback indicating trunk movement, and scapular elevation through non-immersive VR; timing: concurrent; frequency: 100%; valence: negative;	Trunk forward lean: fb < ctr (29.9% and 13.3% reductions); Trunk rotation: fb < ctr (19.8% and 4.2% reductions);
Da-Silva et al^34^; Pilot RCT comparing feedback (fb) versus no feedback (ctr)	feedback group:n = 12 age: 73 [65-80];control group: n = 16 age: 69 [61-80] men and women	Hemorrhagic and ischemic (anterior circulation, posterior circulation, and lacunar infarcts); Acute and early subacute	Median NIHSS: 4 (fb), 5 (ctr); Median star cancellation test for neglect (max 54): 53 (fb), 52 (ctr)	Participants wore wearable on wrist during daily life and were provided a list of motor activities to practice daily; Dose: Watch worn 12 h/d during 4 wk	Haptic vibration if activity in previous 60 min fell below personalized target. Visual feedback about activity level on demand. Timing: summary; Frequency: intermittent; Valence: negative, neutral	No statistical analysis performed
Douglass-Kirk et al^50^; Experiment with crossover design comparing feedback (fb) vs no feedback (ctr)	N = 20; Age: 53 [19-72]Men and women	Unspecified type and location; Chronic	FM-UE: 25 [13-44] MoCA: 25 [14-30];	Repeatedly reaching forward to touch a button on a table, then backward, while seated and listening to self-selected music.; Dose: 50 movements per condition.	Musical played only while movements were performed without compensatory shoulder abduction, shoulder elevation, or trunk flexion; Timing: concurrent; Frequency: 100% Valence: positive	Duration of compensatory movements: Fb (19.3%) < ctr (39.4%)
Fruchter et al^52^; Experiment with crossover design comparing feedback (fb) vs no feedback (ctr)	N = 11; Age: 63 [45-77];Men and women	Hemorrhagic and ischemic; unspecified location; Subacute and chronic	FM-UE: 45 [30-54]; MMSE ≥ 24 or MoCA ≥ 23; Exclusion: neglect, aphasia	In each trial, participants arranged 6 cups onto targets on a table; Dose: 12 trials per condition;	Verbal feedback from computer about compensatory trunk flexion, scapular elevation, or elbow extension; Timing: Terminal (3-4 s delay); Frequency: based on decision tree algorithm; Valence: pos. and neg.	No differences between conditions in number movements with compensation.
Schwerz de Lucena et al^39^; RCT comparing feedback (FB) versus no feedback (CTR)	feedback group:n = 10 age: 56 control group: n = 10 age: 58; men and women	Hemorrhagic and ischemic; unspecified location; Chronic	FM-UE: 41 (CTR), 40 (FB);	Participants were given a wearable device that measured hand movements during daily life. During “hand sprint” game, the device encouraged 30s burst of hand movements. Dose: Participants wore the device for 17.2 (FB) and 16.1 (CTR) d, for 11.2 (FB) and 10.1 (CTR) h/d on average.	The device displayed cumulative number of hand movements each day. An emoji indicated progress relative to daily goal and goal during “hand sprint” game; Timing: concurrent, summary; Frequency: intermittent; Valence: positive, negative, neutral.	Slope for change in hand use over time (hand activity counts/d): Fb (9.0) > ctr (4.9)
Valdes et al^56^; Randomized crossover trial comparing visual (VIS) vs force (FRC) feedback	N = 15; Age = 64 [45-83); Men and women	Hemorrhagic and ischemic; unspecified location; Chronic	FM-UE: 39.6 [15-59];	Bimanual forward planar reaches while holding robotic manipulandums in each hand. Dose: 60 movement repetitions per condition.	The amount of trunk displacement was indicated either by a visual cursor filling with red (VIS), or resistive haptic forces (FRC). Timing: concurrent; Frequency: 100%; Valence: negative	Trunk displacement (mm): VIS (69.8) < baseline (119.2) FRC (68.7) < baseline (119.2) No difference between VIS and FRC
Valdes et al^57^; Randomized crossover trial comparing visual and force (VF) vs visual, force, and game score (VFS) feedback	N = 14; Age = 58 [36-70); Men and women	Hemorrhagic and ischemic; unspecified location; Chronic	FM-UE: 33.6;	Bimanual forward planar reaches while holding robotic manipulandums in each hand. Dose: 60 movement repetitions per condition.	In both groups, the amount of trunk displacement was indicated by a visual cursor filling with red, and resistive haptic forces. In VFS, a score from 0 to 100 was inversely related to trunk compensation. Timing: concurrent; Frequency: 100%; Valence: positive, negative	Trunk displacement (mm): VF (51.6) < baseline (101.4) SVF (55.1) < baseline (101.4) No difference between VF and SVF

Abbreviations: FM-UE, Fugyl-Meyer Upper Extremity Score (max: 66); MMSE: mini mental status examination; VR, virtual reality; NIHSS, National Institute of Health Stroke Scale; MoCa: Montreal Cognitive Assessment (max: 30);

Overall, the studies reviewed here found that feedback consistently produced reductions in compensation with large effect sizes, with group average reductions in the duration and magnitude of compensatory movements ranging between about 40% to 50%. The effects of feedback on compensation did not depend on variations in the feedback such as the sensory modality or the addition of gamified scoring. Only 1 study, Fruchter et al,^52^ did not find reliable reductions in compensation due to feedback. This study differed from the others in that it involved a task with more complex cognitive and motor demands. Furthermore, feedback was manually controlled by a therapist, which possibly resulted in delayed and imprecise feedback compared to automated systems.

Schwerz de Lucena et al^39^ tested the effects of feedback from a wearable device designed to encourage use of the hemiparetic hand during daily life. They reported greater increases in the amount of hand use over time in a group receiving feedback, compared to a group wearing the device without feedback. However, they found no differences between groups in clinical outcomes nor hand use at a 3-month follow up assessment.

### Characteristics of Feedback

#### Modality of Feedback

Sixteen studies included non-verbal auditory feedback,^32,36-38,40-43,45-48,50,54,58,59^ 18 studies included non-verbal visual feedback,^30,31,33,35-37,39-41,43,44,46,47,53,55-57,58^ 4 studies included haptic feedback,^34,49,56,57^ and 4 studies included verbal feedback either by therapists or automated systems.^30,31,46,51,52^ Only 1 study was designed to compare the effects of feedback with similar informational content delivered through different sensory modalities, finding that visual and haptic feedback produced similar reductions in compensatory movements.^56^

#### Valence of Feedback

Nineteen studies included feedback indicating unsuccessful task performance (negative valence),^32,34,36,39,41-46,48,49,52,54-59^ 14 studies included feedback indicating successful task performance (positive valence),^36,38,39,41,43,44,47,48,50,52,55,57-59^ and 8 delivered performance feedback with neutral valence, such as a point score indicating performance that was not explicitly framed as good or bad.^30,31,33,34,38,44,47,51,53^ Only 1 study directly compared feedback with positive versus negative valence, finding that while both positive (reward) and negative (punishment) feedback improved the acquisition of motor learning, only positive feedback improved retention.^43^

#### Timing of Feedback

Fifteen studies involved continuous feedback concurrent with movements,^30-33,37,39,42,45,46,48-50,54-57^ and 15 studies involved terminal feedback at the end of movements.^30,31,33,35,36,38,41,43,44,47,51-53,55,58,59^ One study did not provide information about the timing of feedback.^40^ Only 2 studies provided information regarding the delay of terminal feedback. Fruchter et al^52^ reported that the delay was approximately 3 to 4 seconds. Widmer et al^43^ intentionally imposed a 1 s delay in the control condition in order to reduce the efficacy of feedback, as it was not possible to eliminate performance feedback entirely in the task. No study specifically varied the timing of feedback while controlling for all other feedback characteristics.

#### Scheduling of Feedback

Twenty-two studies provided feedback, or a possibility of feedback, for every movement.^32,33,36-38,41,43-51,53-59^ Three studies provided intermittent feedback with reducing frequency over time (faded feedback).^31,33,42^ Two studies provided intermittent summary feedback regarding the amount of use of the upper extremity during daily life.^34,39^ In 1 study, a decision tree algorithm determined whether feedback was delivered after each movement.^52^ Two studies did not provide information regarding the frequency of feedback.^35,40^ No study specifically varied the scheduling of feedback while controlling for other factors.

#### Elements of Additional Motivational Salience

Two studies used monetary reward linked to performance feedback,^43,44^ and 1 study used monetary punishment.^44^ Eleven studies used gamified scoring.^35-41,43,47,57,59^ Five studies used virtual or augmented reality.^36,40,41,46,59^ Two studies delivered performance feedback in the form of musical stimuli.^48,50^ One study used social comparison tied to performance feedback in the form of a high score list.^38^ Four studies directly compared feedback conditions with different elements of motivational salience that were otherwise similar, including monetary incentives,^43,44^ gamified scoring,^41,43,57^ and complex multisensory stimuli.^41,43^ These studies found that the addition of these motivational elements can improve both motor learning and recovery. While the addition of simple game scoring and virtual reality stimuli to feedback produced moderate learning-related improvements in reaching kinematics,^41^ a more elaborate gamified experience coupled with monetary reward during reaching practice produced clinical gains well above established minimum clinically important differences.^43^

## Discussion

We identified 29 studies that tested the effects of feedback on upper limb motor outcomes in stroke. Nearly all (26/29) of the studies reviewed here were published after previous reviews on the topic.^26,27^ Most of the studies included in these previous reviews didn’t isolate the effects of feedback, as they lacked control conditions in which participants performed similar activities with different feedback, and therefore were not included in the current review.

Beneficial effects of feedback were reported across a range of endpoints in clinical trials spanning all 3 domains of the ICF model. These results are promising in that they indicate that feedback may have the potential to confer benefits on clinically meaningful and valid measures. However, the mechanisms by which feedback produced benefits were ambiguous in the majority of studies, as feedback during rehabilitation could improve coarse clinical measures in multiple, very different ways. For example, feedback could improve dexterity by enhancing the consolidation of motor skill learning between rehabilitation sessions,^18,23^ or it could lead to greater peripheral muscle adaptations by motivating increased effort during rehabilitation exercises.^65^ Either mechanism could produce benefits in clinical assessments, which are often sensitive to both dexterity and strength.^66^

Applications of feedback are likely to be more effective if their design and evaluation are targeted to specific neurobehavioral mechanisms. We found that besides clinical measures, the effects of feedback related to more specific behavioral measures could be classified according to 3 distinct concepts: motor learning, performance, and action selection. We mapped the current evidence for effects of feedback on upper extremity function post-stroke onto these distinct concepts in order to better understand what is currently known, and what questions remain outstanding.

### Effects of Feedback on Motor Learning

The use of feedback to improve motor learning is often cited as a fundamental principle of neurorehabilitation.^2,27,67^ We identified 4 studies that reported effects of feedback on motor learning. Learning improvements due to feedback were mostly specific to the movement parameters that were relayed by feedback (ie, endpoint accuracy,^31^ shoulder and elbow motion,^30^ and movement trajectories^44,45^). Therefore, the information content of feedback should be carefully selected to convey aspects of movement that are clinically important. Ongoing work to establish valid and responsive metrics for quality of movement post-stroke may be informative in determining which parameters should be relayed to patients through feedback.^68,69^

A key question is whether feedback-mediated motor learning during specific tasks can generalize beyond the training context to produce meaningful reductions in impairment or activity limitations. Only 1 study identified here directly tested generalization. They found that improved movement kinematics due to feedback when training reaches toward 1 target generalized to untrained reaches toward a different target.^30,31^ However, this is a rather limited form of generalization, and its clinical significance is unclear. Two studies tested the effects of feedback on both motor learning and clinical outcomes. In both cases, feedback manipulations improved motor learning, but these improvements did not generalize to differences in clinical measures.^30,31,41^ We recommend that future work investigating the effects of feedback on motor learning post-stroke also includes generalization tests that approximate activities of daily living.

### Effects of Feedback on Performance

The introduction of feedback can lead to immediate improvements in the vigor and accuracy of movements.^22^ We identified evidence for feedback-mediated performance benefits on movement amplitude,^49^ speed,^49,51,53^ accuracy,^53,54^ and smoothness.^55^ As was the case for motor learning, performance tended to improve on variables that were directly related to the content of the feedback. Performance improvements during rehabilitation may benefit long-term outcomes. However, improvements may not persist beyond rehabilitation if performance becomes reliant on extrinsic feedback.^11,12,70^ Future work is needed to understand how short-term performance gains due to feedback during stroke rehabilitation translate to longer-term outcomes such as learning and recovery.

Effects of feedback on performance were mainly reported for simple feedback indicating movement execution relative to task goals, compared to no feedback.^49,53-55^ No studies tested whether elements of motivational salience coupled with feedback improved motor performance. It is well established that rewarding feedback can increase movement vigor by motivating physical effort.^22,65,71^ Rewarding features such as gamification are common in neurorehabilitation technologies, and may have the potential to promote increases in the intensity of rehabilitation, which is a key factor for recovery.^72,73^ Future research should therefore investigate whether elements of motivational salience tied to feedback can be designed to produce sustained increases in rehabilitation intensity.

### Effects of Feedback on Action Selection

Feedback could promote recovery by encouraging stroke survivors to practice impaired movement patterns instead of compensatory ones. The studies reviewed here show that various types of feedback can effectively reduce the compensation during reaching.^46,50,56,57^ Additionally, 2 pilot RCTs reported that feedback designed to discourage compensatory trunk movements during rehabilitation improved clinical outcomes.^32,42^ Larger randomized controlled trials are needed to confirm whether feedback to reduce compensation during therapy can result in clinically meaningful enhancements of recovery.

Besides feedback, physical trunk restraint has often been used to prevent compensation during rehabilitation.^74^ Although effective in preventing compensatory trunk flexion, physical trunk restraint doesn’t address other forms of compensation involving the scapula and shoulder.^74^ Feedback could provide a more versatile solution as it could in principle be targeted to any compensatory movement pattern. Furthermore, the use of feedback could have lasting benefits if reductions in compensation are retained as long-term habits that generalize to activities of daily living. Future research is needed to test the retention and generalization of feedback-mediated reductions in compensation.

Two studies involved feedback from wearable devices to promote increased use of the affected upper extremity during daily life. One was a pilot study that did not report any statistical analysis of its outcomes, and the other found that feedback resulted in modest increases in hand use over time that did not correspond to any benefits on clinical outcomes. Therefore, it is not yet clear whether feedback can promote recovery post-stroke by promoting increased use of the more affected limb.

### Do the Characteristics of Feedback Influence Motor Outcomes Post-Stroke?

The sensory modality, valence, timing, and scheduling of feedback varied widely across studies. However, the heterogeneity in interventions and outcomes makes it difficult to draw any conclusions regarding the effects of these feedback characteristics. Only a few studies performed direct, controlled comparisons between different types of feedback. The addition of monetary rewards, gamified scoring, and complex multisensory sensory stimuli coupled with positive performance feedback was shown to benefit multiple forms of motor learning and clinical outcomes of rehabilitation.^41,43,44^ These findings suggest that the well-established benefits of reward during motor learning in healthy populations also extend to stroke survivors, and that these benefits can potentially be leveraged to improve the effectiveness of rehabilitation.

One study found that motor performance was improved when verbal feedback was formulated to promote an external, rather than an internal, focus of attention. This is in line with extensive research indicating that motor performance and learning is facilitated by a focus on movement outcomes, rather than the details of movement execution.^75^ A unique problem in stroke, however, is that the most efficient way to achieve successful movement outcomes is generally to use compensatory movement patterns that are counterproductive to recovery in the long term.^25^ Indeed, another study found that patients learned to improve reach precision when given feedback about the movement outcome, but that quality of movement only improved through feedback about movement execution.^30,31^ An optimal strategy may be to first use feedback to train the desired patterns of movement execution to a high level of automaticity, so that the focus of attention can then be shifted toward the movement outcome.

### Do Clinical Characteristics Interact With the Effects of Feedback?

The processing of motor feedback depends on specific neural structures as well as cognitive and perceptual capacities that may be compromised to varying degrees by stroke.^9,10,76-78^ The majority of studies excluded participants with cognitive impairments that are common in stroke survivors, limiting the generalizability of the results. Future work is needed to better understand how specific cognitive impairments or patterns of brain damage might impair the use of feedback, and how feedback can be designed to overcome these potential barriers.

### Neural Mechanisms of Reward-Based Motor Learning

In theories of biological reinforcement learning, the production of rewarded behaviors is thought to be strengthened through neuroplasticity in the striatum and frontal cortical areas mediated by the release of dopamine.^79^ Dopamine signaling is both necessary and sufficient for various forms of motor learning and motor cortical plasticity.^81-87^ Rewards linked to good motor performance have been shown to improve both the rate and retention of motor learning,^18-20,87^ possibly by affecting activity in cortico-striatal network.^24,89^ Interestingly, positive performance feedback alone may act as a form of intrinsic reward which promotes both motor learning and neural activation in reward processing brain regions.^7,90-92^ Future research should ask whether the benefits of performance feedback and reward during stroke rehabilitation depend on cortico-striatal networks, and whether they are impacted by damage to these regions.^93^

### Next Steps

Although the results reviewed here show that performance feedback is promising as a tool to improve recovery, larger randomized controlled trials are clearly needed. However, we suggest that it may be too early to embark on such studies, as a systematic framework for designing feedback systems is still lacking. This reflects a general lack of clarity on the “active ingredients” of rehabilitation. The field should first seek to identify the mechanisms of training-induced recovery, then ways to formulate feedback that increase the exposure to “active ingredients” of therapy through its effects on motor learning, performance, and action selection. Subsequently, it will be necessary to systematically vary the characteristics of feedback in controlled studies to determine how its effects can be optimized. Finding new strategies to enhance the rewarding effects of performance feedback may be particularly important.

### Limitations of the Existing Evidence

The clinical trials reviewed here mostly had small sample sizes and were highly heterogeneous in their treatment protocols and outcome measures. Conclusive evidence for clinical benefits related to feedback during therapy will require larger trials and/or more standardized treatment and study protocols.

## Conclusions

Feedback can affect motor performance, motor learning, and action selection post-stroke in ways that may benefit rehabilitation outcomes. The inclusion of more granular behavioral measures in clinical trials could lead to a better understanding of how these specific processes relate to the effects of feedback on clinical endpoints. The use of salient and rewarding stimuli coupled with positive performance feedback may benefit motor learning and recovery post-stroke. Otherwise, there is very limited direct evidence to support the use of specific feedback characteristics at this time, and more research is needed in this area.

## References

[1] Anderson DI Magill RA Mayo AM Steel KA . Enhancing motor skill acquisition with augmented feedback. In: Hodges NJ Williams MA , eds. Skill Acquisition in Sport. 3rd ed. Routledge; 2019:3-19.

[2] Levin MF Demers M. Motor learning in neurological rehabilitation. Disabil Rehabil. 2021;43(24):3445-3453. doi:10.1080/09638288.2020.1752317 32320305

[3] Kim GJ Parnandi A Eva S Schambra H. The use of wearable sensors to assess and treat the upper extremity after stroke: a scoping review. Disabil Rehabil. 2021;44(20):6119-6138. doi:10.1080/09638288.2021.1957027 34328803 PMC9912423

[4] Laver KE Lange B George S Deutsch JE Saposnik G Crotty M. Virtual reality for stroke rehabilitation. Cochrane Database Syst Rev. 2017;11:CD008349. doi:10.1002/14651858.CD008349.pub4 PMC6485957 29156493

[5] Tosto-Mancuso J Tabacof L Herrera JE , et al. Gamified neurorehabilitation strategies for post-stroke motor recovery: challenges and advantages. Curr Neurol Neurosci Rep. 2022;22(3):183-195. doi:10.1007/s11910-022-01181-y 35278172 PMC8917333

[6] Shadmehr R Smith MA Krakauer JW. Error correction, sensory prediction, and adaptation in motor control. Annu Rev Neurosci. 2010;33:89-108. doi:10.1146/annurev-neuro-060909-153135S 20367317

[7] Lohse K Miller M Bacelar M Krigolson O . Errors, rewards, and reinforcement in motor skill learning. In: Hodges NJ Williams AM , eds. Skill Acquisition in Sport. 3rd ed. Routledge; 2019:39-60.

[8] Chen X Holland P Galea JM. The effects of reward and punishment on motor skill learning. Curr Opin Behav Sci. 2018;20:83-88. doi:10.1016/j.cobeha.2017.11.011

[9] Palidis DJ Cashaback JGA Gribble PL. Neural signatures of reward and sensory error feedback processing in motor learning. J Neurophysiol. 2019;121(4):1561-1574. doi:10.1152/jn.00792.2018 30811259 PMC6485737

[10] Uehara S Mawase F Celnik P. Learning similar actions by reinforcement or sensory-prediction errors rely on distinct physiological mechanisms. Cerebral Cortex. 2018;28(10):3478-3490. doi:10.1093/cercor/bhx214 28968827 PMC6887949

[11] Ronsse R Puttemans V Coxon JP , et al. Motor learning with augmented feedback: modality-dependent behavioral and neural consequences. Cerebral Cortex. 2011;21(6):1283-1294. doi:10.1093/cercor/bhq209 21030486

[12] Sigrist R Rauter G Riener R Wolf P. Augmented visual, auditory, haptic, and multimodal feedback in motor learning: a review. Psychon Bull Rev. 2013;20(1):21-53. doi:10.3758/s13423-012-0333-8 23132605

[13] Brudner SN Kethidi N Graeupner D Ivry RB Taylor JA. Delayed feedback during sensorimotor learning selectively disrupts adaptation but not strategy use. J Neurophysiol. 2016;115(3):1499-1511. doi:10.1152/jn.00066.2015 26792878 PMC4808111

[14] Vassiliadis P Lete A Duque J Derosiere G. Reward timing matters in motor learning. iScience. 2022;25(5):104290. doi:10.1016/j.isci.2022.104290 35573187 PMC9095742

[15] McKay B Hussien J Vinh MA Mir-Orefice A Brooks H Ste-Marie DM. Meta-analysis of the reduced relative feedback frequency effect on motor learning and performance. Psychol Sport Exerc. 2022;61:102165. doi:10.1016/j.psychsport.2022.102165

[16] Kooij K van der Wijdenes LO Rigterink T Overvliet KE Smeets JBJ . Reward abundance interferes with error-based learning in a visuomotor adaptation task. PLoS One. 2018;13(3):e0193002. doi:10.1371/journal.pone.0193002 PMC5841744 29513681

[17] Krause D Koers T Maurer LK. Valence-dependent brain potentials of processing augmented feedback in learning a complex arm movement sequence. Psychophysiology. 2020;57(3):e13508. doi:10.1111/psyp.13508 31777970

[18] Abe M Schambra H Wassermann EM Luckenbaugh D Schweighofer N Cohen LG. Reward improves long-term retention of a motor memory through induction of offline memory gains. Curr Biol. 2011;21(7):557-562. doi:10.1016/j.cub.2011.02.030 21419628 PMC3075334

[19] Galea JM Mallia E Rothwell J Diedrichsen J. The dissociable effects of punishment and reward on motor learning. Nat Neurosci. 2015;18(4):597-602. doi:10.1038/nn.3956 25706473

[20] Vassiliadis P Derosiere G Dubuc C , et al. Reward boosts reinforcement-based motor learning. iScience. 2021;24(7):102821. doi:10.1016/j.isci.2021.102821 34345810 PMC8319366

[21] Wulf G Shea C Lewthwaite R. Motor skill learning and performance: a review of influential factors. Med Educ. 2010;44(1):75-84. doi:10.1111/j.1365-2923.2009.03421.x 20078758

[22] Codol O Holland PJ Manohar SG Galea JM. Reward-based improvements in motor control are driven by multiple error-reducing mechanisms. J Neurosci. 2020;40(18):3604-3620. doi:10.1523/JNEUROSCI.2646-19.2020 32234779 PMC7189755

[23] Wilkinson L Steel A Mooshagian E , et al. Online feedback enhances early consolidation of motor sequence learning and reverses recall deficit from transcranial stimulation of motor cortex. Cortex. 2015;71:134-147. doi:10.1016/j.cortex.2015.06.012 26204232 PMC4575846

[24] Widmer M Ziegler N Held J Luft A Lutz K . Chapter 13 - Rewarding feedback promotes motor skill consolidation via striatal activity. In: Studer B Knecht S , eds. Progress in Brain Research, vol 229. Motivation. Elsevier; 2016:303-323. doi:10.1016/bs.pbr.2016.05.006 27926445

[25] Jones TA. Motor compensation and its effects on neural reorganization after stroke. Nat Rev Neurosci. 2017;18(5):267-280. doi:10.1038/nrn.2017.26 28331232 PMC6289262

[26] Subramanian SK Massie CL Malcolm MP Levin MF. Does provision of extrinsic feedback result in improved motor learning in the upper limb poststroke? A systematic review of the evidence. Neurorehabil Neural Repair. 2010;24(2):113-124. doi:10.1177/1545968309349941 19861591

[27] Molier BI Van Asseldonk EHF Hermens HJ Jannink MJA . Nature, timing, frequency and type of augmented feedback; does it influence motor relearning of the hemiparetic arm after stroke? A systematic review. Disabil Rehabil. 2010;32(22):1799-1809. doi:10.3109/09638281003734359 20345249

[28] Israely S Carmeli E. Error augmentation as a possible technique for improving upper extremity motor performance after a stroke: a systematic review. Top Stroke Rehabil. 2016;23(2):116-125. doi:10.1179/1945511915Y.0000000007 26382572

[29] Liu LY Li Y Lamontagne A. The effects of error-augmentation versus error-reduction paradigms in robotic therapy to enhance upper extremity performance and recovery post-stroke: a systematic review. J Neuroeng Rehabil. 2018;15(1):65. doi:10.1186/s12984-018-0408-5 29973250 PMC6033222

[30] Cirstea MC Levin MF. Improvement of arm movement patterns and endpoint control depends on type of feedback during practice in stroke survivors. Neurorehabil Neural Repair. 2007;21(5):398-411. doi:10.1177/1545968306298414 17369514

[31] Cirstea CM Ptito A Levin MF. Feedback and cognition in arm motor skill reacquisition after stroke. Stroke. 2006;37(5):1237-1242. 16601218 10.1161/01.STR.0000217417.89347.63

[32] Bang DH. Effect of modified constraint-induced movement therapy combined with auditory feedback for trunk control on upper extremity in subacute stroke patients with moderate impairment: randomized controlled pilot trial. J Stroke Cerebrovasc Dis. 2016;25(7):1606-1612. 27062417 10.1016/j.jstrokecerebrovasdis.2016.03.030

[33] Carey JR Durfee WK Bhatt E , et al. Comparison of finger tracking versus simple movement training via telerehabilitation to alter hand function and cortical reorganization after stroke. Neurorehabil Neural Repair. 2007;21(3):216-232. 17351083 10.1177/1545968306292381

[34] Da-Silva RH Moore SA Rodgers H , et al. Wristband accelerometers to motiVate arm exercises after stroke (WAVES): a pilot randomized controlled trial. Clin Rehabil. 2019;33(8):1391-1403. 30845829 10.1177/0269215519834720

[35] Fluet GG Qiu Q Patel J Cronce A Merians AS Adamovich SV. Autonomous use of the home virtual rehabilitation system: a feasibility and pilot study. Games health J. 2019;8(6):432-438. doi:10.1089/g4h.2019.0012 31769724 PMC7133442

[36] Henrique PPB Colussi EL De Marchi ACB . Effects of exergame on patients’ balance and upper limb motor function after stroke: a randomized controlled trial. J Stroke Cerebrovasc Dis. 2019;28(8):2351-2357. doi:10.1016/j.jstrokecerebrovasdis.2019.05.031 31204204

[37] Park JS Lee G Choi JB Hwang NK Jung YJ. Game-based hand resistance exercise versus traditional manual hand exercises for improving hand strength, motor function, and compliance in stroke patients: a multi-center randomized controlled study. NeuroRehabilitation. 2019;45(2):221-227. doi:10.3233/NRE-192829 31498145

[38] Popovic MD Kostic MD Rodic SZ Konstantinovic LM. Feedback-mediated upper extremities exercise: increasing patient motivation in poststroke rehabilitation. BioMed Res Int. 2014;2014:520374. 24991557 10.1155/2014/520374 PMC4060770

[39] Schwerz de Lucena D Rowe JB Okita S Chan V Cramer SC Reinkensmeyer DJ. Providing real-time wearable feedback to increase hand use after stroke: a randomized, controlled trial. Sensors. 2022;22(18):14. 10.3390/s22186938 PMC9505054 36146287

[40] Shin JH Kim MY Lee JY , et al. Effects of virtual reality-based rehabilitation on distal upper extremity function and health-related quality of life: a single-blinded, randomized controlled trial. J Neuroengineering Rehabil. 2016;13(101232233):17. doi:10.1186/s12984-016-0125-x PMC4765099 26911438

[41] Subramanian SK Lourenco CB Chilingaryan G Sveistrup H Levin MF. Arm motor recovery using a virtual reality intervention in chronic stroke: randomized control trial. Neurorehabil Neural Repair. 2013;27(1):13-23. 22785001 10.1177/1545968312449695

[42] Thielman G. Rehabilitation of reaching poststroke: a randomized pilot investigation of tactile versus auditory feedback for trunk control. J Neurol Phys Ther. 2010;34(3):138-144. 20716988 10.1097/NPT.0b013e3181efa1e8

[43] Widmer M Held JPO Wittmann F , et al. Reward during arm training improves impairment and activity after stroke: a randomized controlled trial. Neurorehabil Neural Repair. 2022;36(2):140-150. 34937456 10.1177/15459683211062898 PMC8796156

[44] Quattrocchi G Greenwood R Rothwell JC Galea JM Bestmann S. Reward and punishment enhance motor adaptation in stroke. J Neurol Neurosurg Psychiatry. 2017;88(9):730-736. doi:10.1136/jnnp-2016-314728 28377451

[45] Maulucci RA Eckhouse RH. Retraining reaching in chronic stroke with real-time auditory feedback. NeuroRehabilitation. 2001;16(3):171-182. 11790902

[46] Cai S Wei X Su E Wu W Zheng H Xie L. Online compensation detecting for real-time reduction of compensatory motions during reaching: a pilot study with stroke survivors. J Neuroeng Rehabil. 2020;17(1):58. 32345335 10.1186/s12984-020-00687-1 PMC7189539

[47] Cameirao MS Smailagic A Miao G Siewiorek DP. Coaching or gaming? Implications of strategy choice for home based stroke rehabilitation. J Neuroeng Rehabil. 2016;13:18. 26921185 10.1186/s12984-016-0127-8 PMC4769516

[48] Chen JL Fujii S Schlaug G. The use of augmented auditory feedback to improve arm reaching in stroke: a case series. Disabil Rehabil. 2016;38(11):1115-1124. 26314746 10.3109/09638288.2015.1076530 PMC4769960

[49] Cruz VT Bento V Ruano L , et al. Motor task performance under vibratory feedback early poststroke: single center, randomized, cross-over, controlled clinical trial. Sci Rep. 2014;4:5670. doi:10.1038/srep05670 25011667 PMC4092335

[50] Douglass-Kirk P Grierson M Ward NS , et al. Real-time auditory feedback may reduce abnormal movements in patients with chronic stroke. Disabil Rehabil. 2022;45(4):613-619. 35238694 10.1080/09638288.2022.2037751

[51] Durham KF Sackley CM Wright CC Wing AM Edwards MG van Vliet P. Attentional focus of feedback for improving performance of reach-to-grasp after stroke: a randomised crossover study. Physiotherapy. 2014;100(2):108-115. 23796803 10.1016/j.physio.2013.03.004

[52] Fruchter D Feingold Polak R Berman S Levy-Tzedek S. Automating provision of feedback to stroke patients with and without information on compensatory movements: a pilot study. Front Hum Neurosci. 2022;16:918804. 36003313 10.3389/fnhum.2022.918804 PMC9393297

[53] Rizzo JR Beheshti M Shafieesabet A , et al. Eye-hand re-coordination: a pilot investigation of gaze and reach biofeedback in chronic stroke. Progress Brain Res. 2019;249:361-374. 10.1016/bs.pbr.2019.04.013 31325995

[54] Secoli R Milot MH Rosati G Reinkensmeyer DJ. Effect of visual distraction and auditory feedback on patient effort during robot-assisted movement training after stroke. J Neuroeng Rehabil. 2011;8:21. 21513561 10.1186/1743-0003-8-21 PMC3104373

[55] Simonsen D Popovic MB Spaich EG Andersen OK. Design and test of a Microsoft Kinect-based system for delivering adaptive visual feedback to stroke patients during training of upper limb movement. Med Biol Eng Comput. 2017;55(11):1927-1935. 28343334 10.1007/s11517-017-1640-z

[56] Valdes BA Schneider AN Van der Loos HFM . Reducing trunk compensation in stroke survivors: a randomized crossover trial comparing visual and force feedback modalities. Arch Phys Med Rehabil. 2017;98(10):1932-1940. 28526482 10.1016/j.apmr.2017.03.034

[57] Valdes BA Van der Loos HFM . Biofeedback vs. game scores for reducing trunk compensation after stroke: a randomized crossover trial. Topics Stroke Rehabil. 2018;25(2):96-113. 10.1080/10749357.2017.1394633 29078743

[58] Zimmerli L Krewer C Gassert R Muller F Riener R Lunenburger L. Validation of a mechanism to balance exercise difficulty in robot-assisted upper-extremity rehabilitation after stroke. J Neuroeng Rehabil. 2012;9:6. 22304989 10.1186/1743-0003-9-6 PMC3286404

[59] Colomer C Llorens R Noe E Alcaniz M. Effect of a mixed reality-based intervention on arm, hand, and finger function on chronic stroke. J Neuroeng Rehabil. 2016;13(1):45. 27169462 10.1186/s12984-016-0153-6 PMC4864937

[60] Lang CE Edwards DF Birkenmeier RL Dromerick AW. Estimating minimal clinically important differences of upper-extremity measures early after stroke. Arch Phys Med Rehabil. 2008;89(9):1693-1700. doi:10.1016/j.apmr.2008.02.022 18760153 PMC2819021

[61] Page SJ Fulk GD Boyne P. Clinically important differences for the upper-extremity Fugl-Meyer Scale in people with minimal to moderate impairment due to chronic stroke. Phys Ther. 2012;92(6):791-798. doi:10.2522/ptj.20110009 22282773

[62] Hsieh YW Wang CH Wu SC Chen PC Sheu CF Hsieh CL. Establishing the minimal clinically important difference of the Barthel index in stroke patients. Neurorehabil Neural Repair. 2007;21(3):233-238. doi:10.1177/1545968306294729 17351082

[63] van der Lee JH Beckerman H Knol DL de Vet HCW Bouter LM. Clinimetric properties of the motor activity log for the assessment of arm use in hemiparetic patients. Stroke. 2004;35(6):1410-1414. doi:10.1161/01.STR.0000126900.24964.7e 15087552

[64] Lin KC Hsieh YW Wu CY Chen CL Jang Y Liu JS. Minimal detectable change and clinically important difference of the wolf motor function test in stroke patients. Neurorehabil Neural Repair. 2009;23(5):429-434. doi:10.1177/1545968308331144 19289487

[65] Summerside EM Shadmehr R Ahmed AA. Vigor of reaching movements: reward discounts the cost of effort. J Neurophysiol. 2018;119(6):2347-2357. doi:10.1152/jn.00872.2017 29537911 PMC6734091

[66] Hadjiosif AM Branscheidt M Anaya MA , et al. Dissociation between abnormal motor synergies and impaired reaching dexterity after stroke. J Neurophysiol. 2022;127(4):856-868. doi:10.1152/jn.00447.2021 35108107 PMC8957333

[67] Maier M Ballester BR Verschure PFMJ . Principles of neurorehabilitation after stroke based on motor learning and brain plasticity mechanisms. Front Syst Neurosci. 2019;13:74. doi:10.3389/fnsys.2019.00074 31920570 PMC6928101

[68] Kwakkel G Van Wegen E Burridge JH , et al. Standardized measurement of quality of upper limb movement after stroke: consensus-based core recommendations from the second stroke recovery and rehabilitation roundtable. Int J Stroke. 2019;14(8):783-791. doi:10.1177/1747493019873519 31510885

[69] Saes M Mohamed Refai MI van Beijnum BJF , et al. Quantifying quality of reaching movements longitudinally post-stroke: a systematic review. Neurorehabil Neural Repair. 2022;36(3):183-207. doi:10.1177/15459683211062890 35100897 PMC8902693

[70] Schmidt RA . Frequent augmented feedback can degrade learning: evidence and interpretations. In: Requin J Stelmach GE , eds. Tutorials in Motor Neuroscience. NATO ASI Series. Springer Netherlands; 1991:59-75. doi:10.1007/978-94-011-3626-6_6

[71] Savoie FA Hamel R Lacroix A Thénault F Whittingstall K Bernier PM. Luring the motor system: impact of performance-contingent incentives on pre-movement beta-band activity and motor performance. J Neurosci. 2019;39(15):2903-2914. doi:10.1523/JNEUROSCI.1887-18.2019 30737309 PMC6462448

[72] Hsieh CY Huang HC Wu DP Li CY Chiu MJ Sung SF. Effect of rehabilitation intensity on mortality risk after stroke. Arch Phys Med Rehabil. 2018;99(6):1042-1048.e6. doi:10.1016/j.apmr.2017.10.011 29108967

[73] Ballester BR Ward NS Brander F Maier M Kelly K Verschure PFMJ . Relationship between intensity and recovery in post-stroke rehabilitation: a retrospective analysis. J Neurol Neurosurg Psychiatry. 2022;93(2):226-228. doi:10.1136/jnnp-2021-326948 34168083 PMC8784991

[74] Pain LM Baker R Richardson D Agur AMR . Effect of trunk-restraint training on function and compensatory trunk, shoulder and elbow patterns during post-stroke reach: a systematic review. Disabil Rehabil. 2015;37(7):553-562. doi:10.3109/09638288.2014.932450 24963941

[75] Chua LK Jimenez-Diaz J Lewthwaite R Kim T Wulf G. Superiority of external attentional focus for motor performance and learning: systematic reviews and meta-analyses. Psycholl Bull. 2021;147(6):618-645. doi:10.1037/bul0000335 34843301

[76] Palidis DJ McGregor HR Vo A MacDonald PA Gribble PL. Null effects of levodopa on reward- and error-based motor adaptation, savings, and anterograde interference. J Neurophysiol. 2021;126(1):47-67. doi:10.1152/jn.00696.2020 34038228

[77] Palidis DJ Fellows LK. Dorsomedial frontal cortex damage impairs error-based, but not reinforcement-based motor learning in humans. Cereb Cortex. 2024;34(1):bhad424. doi:10.1093/cercor/bhad424 37955674

[78] Sidarta A van Vugt FT Ostry DJ. Somatosensory working memory in human reinforcement-based motor learning. J Neurophysiol. 2018;120(6):3275-3286. doi:10.1152/jn.00442.2018 30354856 PMC6337046

[79] Schultz W. Neuronal reward and decision signals: from theories to data. Physiol Rev. 2015;95(3):853-951. doi:10.1152/physrev.00023.2014 26109341 PMC4491543

[80] Beeler JA Cao ZFH Kheirbek MA , et al. Dopamine-dependent motor learning: insight into levodopa’s long-duration response. Ann Neurol. 2010;67(5):639-647. doi:10.1002/ana.21947 20437561 PMC3129617

[81] Hosp JA Pekanovic A Rioult-Pedotti MS Luft AR. Dopaminergic projections from midbrain to primary motor cortex mediate motor skill learning. J Neurosci. 2011;31(7):2481-2487. doi:10.1523/JNEUROSCI.5411-10.2011 21325515 PMC6623715

[82] Molina-Luna K Pekanovic A Röhrich S , et al. Dopamine in motor cortex is necessary for skill learning and synaptic plasticity. PLoS One. 2009;4(9):e7082. doi:10.1371/journal.pone.0007082 PMC2738964 19759902

[83] Athalye VR Santos FJ Carmena JM Costa RM. Evidence for a neural law of effect. Science. 2018;359(6379):1024-1029. doi:10.1126/science.aao6058 29496877

[84] Pekny SE Izawa J Shadmehr R. Reward-dependent modulation of movement variability. J Neurosci. 2015;35(9):4015-4024. doi:10.1523/JNEUROSCI.3244-14.2015 25740529 PMC4348194

[85] Flöel A Breitenstein C Hummel F , et al. Dopaminergic influences on formation of a motor memory. Ann Neurol. 2005;58(1):121-130. doi:10.1002/ana.20536 15984008

[86] Vitrac C Nallet-Khosrofian L Iijima M Rioult-Pedotti MS Luft A. Endogenous dopamine transmission is crucial for motor skill recovery after stroke. IBRO Neurosci Rep. 2022;13:15-21. 35707766 10.1016/j.ibneur.2022.05.008 PMC9189999

[87] Dayan E Averbeck BB Richmond BJ Cohen LG. Stochastic reinforcement benefits skill acquisition. Learn Mem. 2014;21(3):140-142. doi:10.1101/lm.032417.113 24532838 PMC3929848

[88] Nikooyan AA Ahmed AA. Reward feedback accelerates motor learning. J Neurophysiol. 2014;113(2):633-646. doi:10.1152/jn.00032.2014 25355957

[89] Vassiliadis P Beanato E Popa T , et al. Non-invasive stimulation of the human striatum disrupts reinforcement learning of motor skills. Nat Hum Behav. 2024;8(8):1581-1598. doi:10.1038/s41562-024-01901-z 38811696 PMC11343719

[90] Izawa J Shadmehr R. Learning from sensory and reward prediction errors during motor adaptation. PLOS Comput Biol. 2011;7(3):e1002012. doi:10.1371/journal.pcbi.1002012 PMC3053313 21423711

[91] Murayama K Matsumoto M Izuma K Matsumoto K. Neural basis of the undermining effect of monetary reward on intrinsic motivation. Proc Natl Acad Sci USA. 2010;107(49):20911-20916. doi:10.1073/pnas.1013305107 21078974 PMC3000299

[92] Chew B Blain B Dolan RJ Rutledge RB. A neurocomputational model for intrinsic reward. J Neurosci. 2021;41(43):8963-8971. doi:10.1523/JNEUROSCI.0858-20.2021 34544831 PMC8549542

[93] Widmer M Lutz K Luft AR. Reduced striatal activation in response to rewarding motor performance feedback after stroke. NeuroImage: Clin. 2019;24:102036. doi:10.1016/j.nicl.2019.102036 31698315 PMC6978223

